# Genotyping and Infection Rate of GBV-C among Iranian HCV- Infected Patients

**Published:** 2010-06-01

**Authors:** Reza Ghanbari, Mehrdad Ravanshad, Seyed Younes Hosseini, Ramin Yaghobi, Kiana Shahzamani

**Affiliations:** 1 Department of Virology, Faculty of Medical Sciences, Tarbiat Modares University, Tehran, Iran; 2 Department of Virology, Shiraz Transplant Research Center, Nemazee Hospital, Shiraz University of Medical Sciences, Shiraz, Iran

**Keywords:** GBV-C, HCV, Prevalence, Genotyping, Iranian, 5’-UTR

## Abstract

**Background and Aims:**

Hepatitis G virus/GB virus-C (HGV/GBV-C) is a newly identified member of the Flaviviridae family. Its clinical significance in chronic hepatitis C infection remains controversial. There is a geographical difference in the distribution of GBV-C in the world. The frequency of GBV-C infection among hepatitis C virus (HCV) infected patients varies. The aim of the current study was to determine the prevalence and genotypes of GBV-C among Iranian patients infected with chronic HCV.

**Methods:**

Infection with GBV-C was surveyed in 71 chronic confirmed hepatitis C infected patients. These samples were collected at the Digestive Disease Research Center (DDRC) of Shariati Hospital, Tehran, Iran from January to October 2007. The 5’-UTR region of GBV-C RNA was detected using a novel in-house touchdown nested reverse transcription polymerase chain reaction (RT-PCR), the products were sequenced and the results were aligned and phylogenically analyzed.

**Results:**

Of the 71 HCV-infected patients, 31 (43.6%) were found positive for GBV-C RNA. Sequencing and phylogenic analysis showed that the samples were Genotype 2 of GBV-C.

**Conclusions:**

It seems that there is a high rate of GBV-C infection among Iranian patients infected with chronic HCV. In comparison with the six reference genotypes, it was observed that all the samples were categorized in Genotype 2 of GBV-C, prevalent in North America, Africa and in European countries.

## Introduction

The GB virus C (GBV-C) and hepatitis G virus (HGV) were independently discovered (1, 2), but it was later determined that they were two isolates of the same virus [3][4]. Since subsequent studies did not find an association between these viruses and hepatitis, most researchers refer to the virus as GBV-C [5]. The GBV-C is a single-stranded, positive-sense RNA virus, a member of the Flaviviridae [1][2]. Based on a comparison between genome organization and sequence homologies, GBV-C is most closely related to human hepatitis C virus (HCV), another member of the Flaviviridae [1][6] However, in contrast to HCV, GBV-C does not appear to be hepatotrophic [7][8]. In fact, GBV-C replicates within the cells of the hemopoietic lineage and lymphocytes [9]. Based on sequence and phylogenetic analysis, it was observed that different genotypes of HCV differ by more than 30%, while the most extreme GBV-C variants differ by only 14% [10]. GBV-C is transmitted through blood transfusion and components [11][12][13].

Epidemiological data suggest that the virus is also spread by sexual and vertical modes of transmission [14][15][16]. GBV-C infection is relatively common and prevalent all over the world. Between 1% and 4% of healthy blood donors have GBV-C RNA detected in their sera [7][17][18]. It is prevalent in high-risk groups, such as people with hemodialysis, hemophiliacs, HCV, human immunodeficiency virus (HIV), and hepatitis B virus (HBV) infected patients, as well as in intravenous drug users.

A number of studies have shown that GBV-C has a profound “protective” influence on HIV and inhibits replication of this virus in vitro, and has been associated with a decreased risk of death and better response to treatment among HIV-positive persons as far as co-infection is concerned[19][20][21][22][23]. Co-infection of GBV-C and HCV is common. Among newly diagnosed cases of blood-borne viral hepatitis in the United States, 18% were found positive for GBV-C, and 80% of these patients were also infected with HCV [24]. Other studies have reported the incidence of GBV-C co-infection with HCV, varying from 11–31.5% [3][25][26][27][28]. The high prevalence of HCV infection and its morbidity, as well as its relation to acute and chronic hepatitis, cirrhosis, and hepatocellular carcinoma is very well documented. As for GBV-C, there is little evidence of morbidity and many doubts about its etiological role in liver diseases in situations of co-infection with HCV or HBV [5][29][30][31]. Most of the studies on GBV-C in Iran have been done on HIV co-infected patients, and the GBV-C infection-rate was reported to be 10.97% [32], 11.3% [33] and 15.5% [34]. In another study, 13% of Iranian hemodialysis patients were also found to be infected with GBV-C [35].

The aim of the current study was to determine the rate of co-infection of GBV-C and HCV, as well as the genotype distribution of GBV-C among Iranian patients infected with chronic HCV.

Based on a variation in the nucleotide sequence of 5’-untranslated region (5’-UTR), GBV-C can be classified into six major genotypes [36][37][38] .The GBV-C genotypes were described as originating in West Africa (Genotype 1), North America and Europe (Genotype 2), East Asia and Japan (Genotype 3), Southeast Asia (Genotype 4), South Africa (Genotype 5), and a recently discovered genotype in Indonesia (Genotype 6). It was also shown that different genotypes have different geographical distribution [36][38][39][40]. Apart from the six genotypes of human GBV-C, a closely related chimpanzee virus “GBV-C tro” was also discovered [41].

Although some studies have reported that Genotypes 3 and 4 are prevalent in most parts of Asia [10][42][43], our study showed that Genotype 2 of GBV-C is most prevalent in HCV-infected patients in Iran [33][35][44], as previously reported in the United Arab of Emirates [45] and Turkey [46]. In the current study, 27 GBV-C isolates from Iranian individuals infected with chronic HCV were sequenced and analyzed to determine the GBV-C genotype.

## Materials and Methods

### Patients

Seventy one Iranian chronic HCV infected patients, in total, who had all been referred to the Digestive Disease Research Center (DDRC) of Shariati Hospital, Tehran, Iran for treatment, were randomly selected for the study during the period January to October 2007. These patients were from different parts of Iran. The study and sampling was approved by Tarbiat Modares University’s Ethics Approval Committee, and informed consent was obtained from the patients. All of the samples were analyzed with Amplicor HCV test v2.0 (Roche Diagnostics, Germany), according to the manufacturer’s instructions, and HCV genotypes were determined based on the HCV genome sequencing of 5’-UTR regions by the restriction fragment length polymorphism (RFLP) method at Keivan Laboratory. The collected sera were stored at -70ºC in an HCV sample bank. The samples consisted of 16 females and 55 males in age ranging from 19 to 57 years old. All of the sera samples were used for RNA extraction, reverse transcription, polymerase chain reaction, and sequencing.

### Amplification of GBV-C sequence

GBV-C RNA was detected by an in-house developed touchdown nested reverse transcription polymerase chain reaction (RT-PCR), using nested primers targeting the 5’-UTR region (Table 1).

Briefly, the total viral RNA was extracted from 140 µl of serum using the QIAamp Viral RNA Kit (Qiagen, Germany), according to the manufacturer’s instructions.Complementary DNA (cDNA) was synthesized from 2 µl of extracted RNA at 25°C for 5 min, at 42°C for 1 h, and at 72°C for 10 min, using Moloney Murine Leukaemia virus reverse transcriptase (M-MuLV-RT), and random hexamers. Each 20 µl RT master mixture contained 1 mmol dNTP, 0.01 mg/ml hexanucleotide, 7.5 U/ml M-MuLV-reverse transcriptase, 1 U/ml RNase inhibitor, and 4 µl 5X RT buffer.

Based on the reference sequences from GenBank, oligonucleotide-specific nested primer pairs were designed to amplify the 5’-untranslated region (5’-UTR), and the final expected product was 188 bp in length Table 1. Two microliters of produced cDNA were used as a template for the first round, and one microliter of the first- round PCR product was used for the second.

For the first PCR round, the PCR master mixture contained: 0.15 pmol/ul primers, 0.2 mmol dNTPs, 1.25U Taq DNA polymerase, 2.5 µl 10× PCR buffer, and 1.5 mmol MgCl2. The reagents for the second PCR round were the same as the ones used in the first round. The total volume per reaction in the two rounds was 25 µl.

Amplification was done in 25 cycles for the first round (94°C for 50 s, 55°C for 40 s, 72°C for 50 s with a final extension at 72°C for 3 min followed by a hold at 4°C) and 30 cycles for the second rounds of PCR (94°C for 40 s, 53°C for 35 s, 72°C for 40 s with a final extension at 72°C for 3 min followed by a hold at 4°C).

All PCR contamination precautions were observed; and negative controls using sera from subjects with no GBV-C markers was obtained from the Iranian Blood Transfusion Organization Research Center. PCR procedure was carefully optimized.

**Figure 1:**
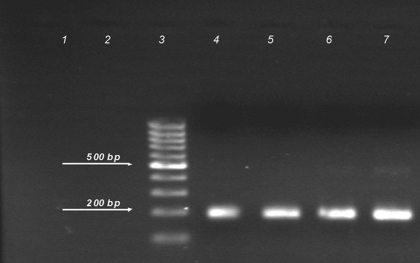
Agarose gel electrophoresis of PCR products. From left: lane 1 and 2 negative samples; lane 3 DNA ladder (100 bp); lane 4 and 5 positive samples (Touchdown RT-PCR); lane 6 and 7 positive samples (Conventional RT-PCR).

To remove small traces of non-specific bands, a touchdown procedure was adopted for the second round (94°C for 50 s, 72°C for 70 s, 8 cycles; 94°C for 40 s, 69°C for 35 s, 72°C for 40 s, 5 cycles; 94°C for 40 s, 67°C for 35 s, 72°C for 40 s, 5 cycles; 94°C for 40 s, 64°C for 35 s, 72°C for 40 s, 8 cycles; 94°C for 40 s, 62°C for 35 s, 72°C for 40 s, 4 cycles; and the final extension 72°C for 2 min followed by a hold at 4°C). The PCR products were analyzed by electrophoresis in a 2% agarose gel and stained with ethidium bromide (Fig. 1). Finally, the product band was selected and purified with the AccuPrep Gel Purification Kit (Bioneer, Korea), and the optical density was measured.

PCR products were directly and bidirectionally sequenced using a BigDye Terminator cycle sequencing kit (Applied Biosystems, CA, USA) with an ABI PRISM 3700 DNA analyzer automated sequencer at Sequence Laboratories Göttingen GmbH (SEQLAB), Germany.

Randomization to either the TACE with gemcitabine plus oxaliplatin combination group (GO group) or the TACE with floxuridine plus oxaliplatin combination group (FO group) was performed without stratification by drawing consecutively numbered sealed envelopes. The protocol was approved by the ethics committee of Fujian Provincial Tumor Hospital. Written informed consent was obtained.

**Table1:** Primer sequences and product length for 5'-UTR region of GBV-C.

**Primer**	**Primer Sequence**	**Product length**
GBV-C-G1 (sense, outer)	5’-GGTCGTAAATCCCGGTCACC-3’	262bp
GBV-C-G2 (anti-sense, outer)	5’-CCCACTGGTCCTTGTCAACT-3’	
GBV-C-G3 (sense, inner)	5’-TAGCCACTATAGGTGGGTCT-3’	188bp
GBV-C-G4 (anti-sense, inner)	5’-ATTGAAGGGCGACGTGGACC-	

### Reference sequences from the database

In total, 30 reference sequences of GBV-C Genotype 1–6 were obtained from GenBank and were used to compare the sequences of the isolates in the study. The accession numbers of the reported sequences and country of the reported sequences were as following: Genotype 1, U36380 (USA), AB013500 (Ghana),AB003291 (Japan); Genotype 2, U44402 (USA), AF121950 (USA), AX338086 (USA), AF081782 (China), AF309966 (Germany), D87255 (Japan), AF031827 (USA), AF031829 (USA), U45966 (USA), AF104403 (France), AB003289 (Japan), D90600 (Japan), AB013501 (Bolivia), U63715 (East Africa), AF172543 (South Africa); Genotype 3, D90601 (Japan), D87262 (Japan), AF006500 (Hong Kong), AB013501 (Japan), AB003293 (Japan), AB003288 (Japan), D87253 (Japan), D87252 (Japan); Genotype 4, AB018667 (Vietnam), AB021287 (Myanmar); Genotype 5, AF131112 (South Africa); Genotype 6, AB003292 (Japan); AF070476 (GBV-C tro).

### Phylogenetic analysis

Phylogenetic analysis was performed, based on GBV-C 5’-UTR nucleotide sequence and related reference sequences. All of the results were edited and first analyzed with Bioedit (Ibis Biosciences, USA), and ClustalX (EMBL-EBI, UK) software for multiple alignments.

**Figure 2:**
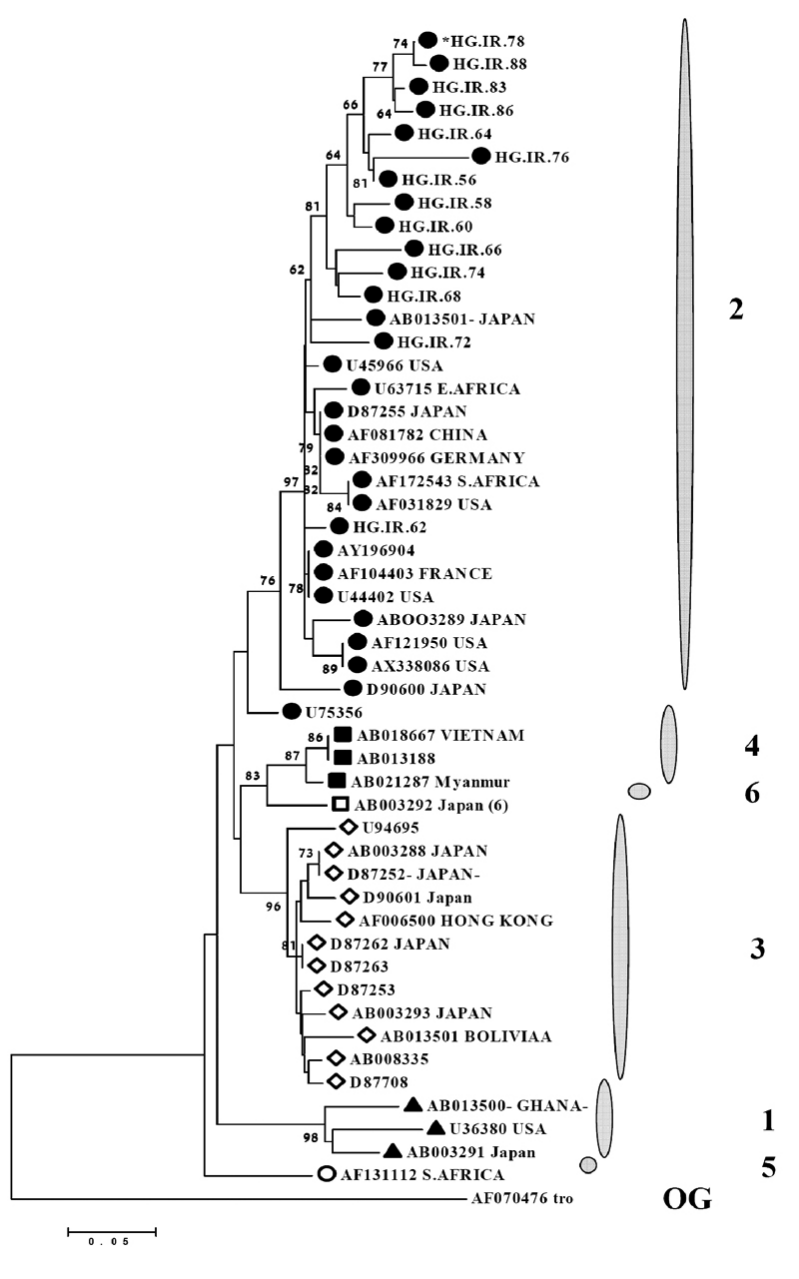
Phylogenetic tree constructed using the Kimura two-parameter matrix and neighbor-joining method, based on 5'-UTR sequence (188nt, residues 163 – 350). References 5’-UTR sequence from GBV-C genomes are identified by their GenBank accession numbers. 12 out of 27 Iranian sequences determined in this study are indicated by the HG.IR prefix. The numbers 1-6 designated GBV-C genotypes.

Genetic distance was estimated using the Kimura-two-parameter matrix [47]. Phylogenetic trees were constructed by the neighbor-joining (NJ) method [48]. Bootstrap resampling and reconstruction were carried out on 100 replicates to ensure consistency. The analysis and calculation of nucleotide differences within and between the isolate sequences were carried out using MEGA4 for Windows (Biodesign Institute, USA) software (Fig. 2).

## Results

GBV-C RNA was detected in 31 (43.6%) out of the 71 Iranian patients with chronic hepatitis C infection. Among the 71 patients with chronic hepatitis C, there were no significant differences in sex (P = 0.393), age (P = 0.260), and HCV genotypes in Iran (1a and 3a) (P = 0.471) in those with and without the GBV-C infection. The samples consisted of 16 (22.5%) females, 6 of them positive for GBV-C RNA, and 55 (77.5%) males, 25 of them positive for GBV-C RNA, with an age range of 19–57 years old (Table 2). Four out of the 31 5’-UTR sequenced were excluded because of unacceptable sequencing results.

The neighbor- joining method based, using a 1000 bootstrap re-sampling replicate tree, on the 27 5’-UTR nucleotide sequences of patients on whom successful amplification and sequencing were performed. Also 30 different reference GBV-C isolates in the GenBank database were included.

The results also showed that Genotype 2 is the most prevalent in infected patients. Based on the results, the Iranian isolates were classified as Genotype 2 with a bootstrap value of >80% based on clustering with previously characterized Genotype 2 isolates and other genotypes Fig.2.

**Table2:** Demographic characteristics of HCV-infected and HCV, GBV-C co-infected patients

**Patients'characteristics**		**Total patients**	**Number of HCV infected patients**	**Number of HCV and GBV-C co-infected patients**
Patients’ Sex	Male	55	30	25
	Female	16	10	6
Patients’ Age Groups (years)	17-27	13	7	6
	28-37	24	13	11
	38-47	22	14	8
	48-57	12	6	6
HCV Genotype	1a	30	19	11
	3a	28	18	10
	1b	5	1	4
	1a + 1b	5	1	4
	1a + 3a	2	-	2
	4	1	1	-

## Discussion

In the current study, GBV-C RNA was detected in 43.6% of Iranian patients infected with chronic HCV. Other studies on GBV-C have reported a relatively lower prevalence. Tanaka et al. demonstrated GBV-C and HCV coinfection in 11% of patients with chronic HCV [27], Alter et al. described this coinfection in 20% [3], Martinol et al. found GBV-C infection in 21% of patients with chronic HCV [26], Feucht et al. found GBV-C infection in 24.4% of HCV-infected patients [25], and Jie Yan et al., Sauleda S et al. and Quiros E et al. described this coinfection, respectively, in 31.5%, 21%, and 19% of patients with chronic HCV [28][49][50]. Al-knawy et al. also demonstrated the GBV-C in 31% of Saudi Arabian HCV-infected patients [51].

The aforementioned studies have shown a wide variation and a difference in prevalence in different geographical areas. The difference between the GBV-C infection-rate, in earlier studies and in the current investigation, may be due to the size of the study group, the methods used to detect GBV-C, the demographic and clinical features of patients, the virulence of different genotypes and strains, and the different patterns of transmission of virus in the world (e.g., blood and blood components, sexual, intravenous injection, etc.). Another preliminary study in Iran reported the prevalence of GBV-C among Iranian chronic HCV-infected patients, about 40% in a smaller study population [52].

The genotypic classification of GBV-C has been extended to six genotypes (1 to 6) based on the 5’-UTR, and/or E2 gene sequences. Earlier studies have suggested that the phylogenetic relationship of GBV-C complete genome sequences can also be reproduced by analysis of the 5’-UTR region [37][40]. The GBV-C genotypes have distinct geographical distributions [40]. Based on the sequence of Residue 163–350 of the 5’-UTR region of Iranian GBV-C isolates, this study found that Genotype 2 seems to be the most common.

Despite the fact that little is known about the GBV-C genotype in the Middle East/Mediterranean region, and despite the lack of significant information on GBV-C sequence(s) from this region, it is reported that Genotype 2 is common. A survey in Turkey has revealed that the predominant genotype is Genotype 2 [46]. In the United Arab Emirates, Abu Odeh et al. have also reported that Genotype 2 was the most prevalent among nationals and non-nationals alike [45].

To date, there is no information about the GBV-C sequences in Iranian patients infected with chronic HCV, and the current study reports the distribution of the GBV-C genotype based on 5’-UTR sequence and phylogenetic analysis in Iran. Twenty-seven GBV-C strains were isolated from Iranian chronic HCV-infected patients and were successfully sequenced. In phylogenetic analysis based on 5’-UTR sequences, Genotype 2 in Iranian isolates was observed with significant bootstrap values. In all phylogenetic trees based on this region, the Iranian isolates were closely related to the strains in North American and European countries.

Similar molecular epidemiology surveys have been conducted for HCV genotyping in Iran. In all of these studies, HCV genotypes have been determined based on HCV genome sequencing of 5’-UTR regions. Their data have shown that different HCV genotypes are distributed in Iran. The most prevalent HCV genotypes were 1a and 3a, and the less prevalent ones were 3b and 4 [53].

In summary, our findings indicated that the prevalence of GBV-C in Iranian patients with chronic HCV is notably high. The high prevalence of GBV-C among Iranians with chronic HCV infection probably reflects similar modes of transmission and risk factors, both intravenously and through exposure to blood and blood products, probably since it is not recommended practice to screen blood units for HGV. The study also showed that Genotype 2 of GBV-C is the most common genotype among Iranian chronic HCV-infected patients.
